# Mitochondrial Damage in Hemoglobinopathies: The Role of Cell-Free mtDNA as a Potential Biomarker

**DOI:** 10.3390/biomedicines14092011

**Published:** 2026-09-07

**Authors:** Sebnem Tekin Neijmann, Asuman Gedikbasi, Feyza Nur Tuncer, Sukru Anil Dogan, Suzin Tatonyan, Mehmet Cihan Balci, Bengi Bekmez, Volkan Karaman, Gulden Fatma Gokcay, Zeynep Karakaş

**Affiliations:** 1 Department of Interdisciplinary Rare Diseases, Institute of Health Sciences, Istanbul University, 34452 Istanbul, Türkiye; sebnemtekin@gmail.com; 2 Department of Pediatric Basic Sciences, Institute of Child Health, Istanbul University, 34452 Istanbul, Türkiye; 3 Department of Genetics, Aziz Sancar Institute of Experimental Medicine, Istanbul University, 34093 Istanbul, Türkiye; ftuncer@istanbul.edu.tr; 4 Life Sciences & Biotechnology Institute, Bezmialem Vakif University, 34820 Istanbul, Türkiye; anil.dogan@bezmialem.edu.tr; 5 Department of Immunology, Institute of Health Sciences, Istanbul University, 34452 Istanbul, Türkiye; suzintatonyan@gmail.com; 6 Division of Nutrition and Metabolism, Department of Pediatrics, Istanbul Faculty of Medicine, Istanbul University, 34452 Istanbul, Türkiye; mcbalci@istanbul.edu.tr (M.C.B.); ghuner@istanbul.edu.tr (G.F.G.); 7 Center for Life Sciences and Technologies, Department of Molecular Biology and Genetics, Bogazici University, 34342 Istanbul, Türkiye; bengibkmz@gmail.com; 8 Department of Medical Genetics, Istanbul Faculty of Medicine, Istanbul University, 34452 Istanbul, Türkiye; volkan.karaman@istanbul.edu.tr; 9 Division of Hematology, Department of Pediatrics, Istanbul Faculty of Medicine, Istanbul University, 34452 Istanbul, Türkiye; zkarakas@istanbul.edu.tr

**Keywords:** hemoglobinopathies, β-thalassemia, sickle cell disease, cell-free mitochondrial DNA

## Abstract

**Background/Objectives:** In hemoglobinopathies such as β-thalassemia and sickle cell disease (SCD), mitochondrial dysfunction has been implicated in chronic oxidative stress and tissue damage. Circulating cell-free mitochondrial DNA (cf-mtDNA) may reflect mitochondrial injury and serve as a biomarker of disease severity and progression. **Methods:** This cross-sectional study included four groups: three patient groups—β-thalassemia (*n* = 36), SCD (*n* = 11), and genetically confirmed mitochondrial disease (positive control, *n* = 13)—and one healthy control group (*n* = 14). Venous blood was collected in EDTA tubes, and plasma was separated by sequential centrifugation. cf-mtDNA was extracted from 200 µL plasma using the Quick-cfDNA™ Serum & Plasma Kit (Zymo Research, Irvine, CA, USA). cf-mtDNA was assessed by qPCR targeting ND1 and COI and analyzed by relative quantification using the 2^−ΔΔCt^ method (Q3 Real-Time PCR System, LongGene). Each sample was run in triplicate. **Results:** cf-mtDNA levels were significantly higher in the β-thalassemia, SCD, and mitochondrial disease groups compared to healthy controls (*p* < 0.001). No significant correlations were observed between cf-mtDNA levels and hemoglobin or oxygenation parameters. **Conclusions:** Elevated relative cf-mtDNA abundance in hemoglobinopathy patients suggests mitochondrial damage, possibly associated with chronic hypoxia and oxidative stress. cf-mtDNA may represent a promising noninvasive biomarker, warranting further longitudinal investigation to evaluate its potential clinical utility.

## 1. Introduction

Hemoglobinopathies represent some of the most prevalent inherited blood disorders worldwide, with beta-thalassemia and sickle cell disease (SCD) being the most prominent clinical subtypes. These conditions are characterized by defective hemoglobin synthesis and a shortened red blood cell lifespan, leading to chronic hemolytic anemia, ineffective erythropoiesis, and systemic oxidative stress [1,2,3]. Over time, these processes can result in tissue hypoxia, inflammatory activation, and progressive target organ damage [3,4].

Recent research has emphasized the role of circulating cell-free mitochondrial DNA (cf-mtDNA), released during cellular injury, as a potent damage-associated molecular pattern (DAMP) that promotes proinflammatory signaling [5]. Due to its histone-free structure, CpG-enriched sequences, and bacterial-like features, cf-mtDNA is recognized by the innate immune system as a danger signal [6,7]. This recognition occurs primarily through pattern recognition receptors (PRRs), such as Toll-like receptor 9 (TLR9), triggering the activation of type I interferons and proinflammatory cytokines [8].

In the context of hemoglobinopathies, multiple pathophysiological factors converge to promote mitochondrial dysfunction, including chronic hemolysis, excessive generation of reactive oxygen species (ROS), and iron overload, all of which compromise mitochondrial membrane integrity and facilitate cf-mtDNA release into the circulation [9].

Recent studies have demonstrated significantly elevated levels of cf-mtDNA in affected individuals compared to healthy controls. These elevations correlate with mitochondrial retention in red blood cells, systemic inflammation, and the formation of neutrophil extracellular traps (NETs) via activation of the cyclic GMP-AMP synthase-stimulator of interferon genes (cGAS–STING) pathway [10,11,12].

The present study aimed to evaluate circulating cf-mtDNA as a potential biomarker of mitochondrial damage in hereditary hemolytic anemias. In this study, cf-mtDNA was assessed by relative quantification, reflecting fold changes in mitochondrial DNA abundance relative to the healthy control group. As an indirect circulating marker released following mitochondrial damage, cf-mtDNA does not itself constitute a direct measurement of damage but may reflect its downstream consequences. Importantly, cf-mtDNA is not intended to replace established diagnostic methods for β-thalassemia or SCD but may provide complementary information on mitochondrial damage and cellular stress that is not directly captured by conventional diagnostic markers. By assessing relative cf-mtDNA abundance in plasma, we sought to explore its potential biological and clinical relevance in hemoglobinopathies.

## 2. Materials and Methods

### 2.1. Study Population

This cross-sectional, observational study included four groups—three patient groups and one healthy control group—recruited between 2022 and 2024 from the hematology outpatient clinic of our hospital: patients with β-thalassemia major (*n* = 36), patients with sickle cell disease (SCD) (*n* = 11), individuals with genetically confirmed mitochondrial diseases (positive control, *n* = 13), and healthy individuals (negative control, *n* = 14). Inclusion criteria were: (i) age 6–45 years; (ii) a confirmed diagnosis of β-thalassemia or sickle cell disease (SCD) based on clinical and laboratory findings; (iii) for β-thalassemia patients, regular transfusion therapy for at least one year, regular chelation therapy, and routine monitoring of organ iron accumulation; (iv) for SCD patients, regular clinical follow-up for disease-related crises and treatment, including monitoring of hydroxyurea-related adverse effects when applicable; and (v) discontinuation of supportive treatments potentially affecting mitochondrial function at least one week before blood sampling. Exclusion criteria were: (i) presence of another chronic disease potentially affecting the study outcomes; (ii) acute infection at the time of sampling; (iii) splenectomy within the preceding month; (iv) non-adherence to chelation therapy in regularly transfused patients; and (v) unwillingness to participate. For the mitochondrial disease and healthy control groups, the presence of another chronic disease or acute infection was considered an exclusion criterion. The mean age and standard deviation for each of the four groups are presented in Table 1; age did not differ significantly between groups (one-way ANOVA, *p* = 0.076). Informed consent was obtained from all participants and/or their legal guardians. The study was approved by the Institutional Ethics Committee (Approval No: 1226342, 22 September 2022).

### 2.2. Sample Collection and Biochemical Measurements

Venous blood samples were collected in the morning, preferably after fasting, into standard EDTA tubes. All patients were clinically stable at the time of blood sampling, with no acute infection; SCD patients had no acute vaso-occlusive crisis. In patients with transfusion-dependent β-thalassemia, blood samples were collected immediately before the scheduled transfusion, approximately three or four weeks after the preceding transfusion. Blood samples were processed within 30 min of venipuncture. Samples showing visible hemolysis were excluded. Complete blood count (CBC) parameters—including hemoglobin (Hb), hematocrit (Hct), and mean corpuscular volume (MCV)—were analyzed using an automated hematology analyzer (Sysmex XR-9000, Sysmex Corporation, Kobe, Japan). For biochemical evaluation, including blood gas measurements (pO_2_, pCO_2_, sO_2_, HCO_3_, and total hemoglobin [ctHb]), venous blood was drawn into heparinized syringes and analyzed within 10–15 min using a blood gas analyzer (Radiometer, Medical ApS, Brønshøj, Denmark) in the clinical biochemistry laboratory. Venous blood gas parameters (pO_2_ and sO_2_) were included as supportive clinical variables to characterize peripheral oxygenation status and were not intended as direct measures of systemic or tissue oxygenation. Plasma samples were obtained by combining plasma from EDTA tubes routinely collected for complete blood count (CBC) monitoring during patients’ clinical visits with plasma from additional EDTA tubes collected specifically for this study. This approach allowed us to increase the plasma volume available for analysis while minimizing the volume of blood drawn from patients, who were already anemic. Following CBC analysis, the same EDTA tubes were assessed for their suitability for cf-mtDNA isolation. Samples with a total volume of less than 3 mL were excluded from the study to ensure adequate DNA yield and consistency across all participants. Plasma samples were prepared by centrifugation at 1600× *g* for 10 min, followed by a second centrifugation at 16,000× *g* for 10 min to eliminate any remaining cellular debris. Supernatants were transferred into sterile microtubes and stored at −80 °C until cf-mtDNA analysis. Repeated freeze–thaw cycles were avoided, and samples were thawed only when required for cf-mtDNA analysis.

### 2.3. Cell-Free mtDNA Extraction and Quantification

Cell-free DNA (cfDNA) was extracted from plasma samples using the Quick-cfDNA™ Serum & Plasma Kit (Zymo Research Corp., Irvine, CA, USA), following the manufacturer’s vacuum-based protocol. Quantification of cfDNA was performed using a NanoDrop ND-2000 spectrophotometer (Thermo Fisher Scientific, Wilmington, DE, USA). Quantitative PCR (qPCR) was carried out on a Q3 Real-Time PCR System (Hangzhou LongGene Scientific Instruments Co., Ltd., Hangzhou, China) using SYBR Green I Master Mix. Each reaction contained 10 ng of cfDNA and primer sets specific for the mitochondrial genes NADH dehydrogenase subunit 1 (ND1) and cytochrome c oxidase subunit 1 (COI). All reactions were run in triplicate, and the mean cycle threshold (Ct) values were calculated for each gene per sample. For relative quantification, mitochondrial targets were normalized against two endogenous nuclear reference genes, GAPDH and B2M. Results are expressed as relative quantification (RQ), calculated using the 2^−ΔΔCt^ method, representing fold changes in mitochondrial DNA abundance relative to the control group.

### 2.4. Statistical Analysis

Statistical analyses were performed using NCSS (Number Cruncher Statistical System) 2007 Statistical Software (LLC, Kaysville, UT, USA). Descriptive statistics were expressed as mean, standard deviation, median, and interquartile range, as appropriate. The distribution of continuous variables was assessed using the Shapiro–Wilk test. Comparisons among multiple groups were performed using one-way analysis of variance (ANOVA) for normally distributed variables and the Kruskal–Wallis test for non-normally distributed variables. Dunn’s multiple-comparison test was used for post hoc subgroup comparisons. Categorical variables were compared using the chi-square test. Receiver operating characteristic (ROC) curve analyses were performed to evaluate the discriminatory performance of the investigated variables for β-thalassemia, sickle cell disease, and mitochondrial disease. The area under the ROC curve (AUC) was calculated, and optimal cut-off values were determined together with sensitivity, specificity, positive predictive value, negative predictive value, and positive likelihood ratio [LR(+)]. Correlations between cf-mtDNA content and laboratory parameters were assessed using correlation coefficients as indicated in the Results. A *p*-value < 0.05 was considered statistically significant.

## 3. Results

### 3.1. Patient Clinical Characteristics

Demographic data of the cases included in the study are presented in Table 1. The general clinical characteristics of subjects in this study are presented in Supplementary Table S1. In general, patients with β-thalassemia and SCD demonstrated the expected trends in clinical laboratory parameters compared with healthy individuals.

### 3.2. cf-mtDNA Are Increased in Patients with β-Thalassemia and SCD

**Figure 1:**
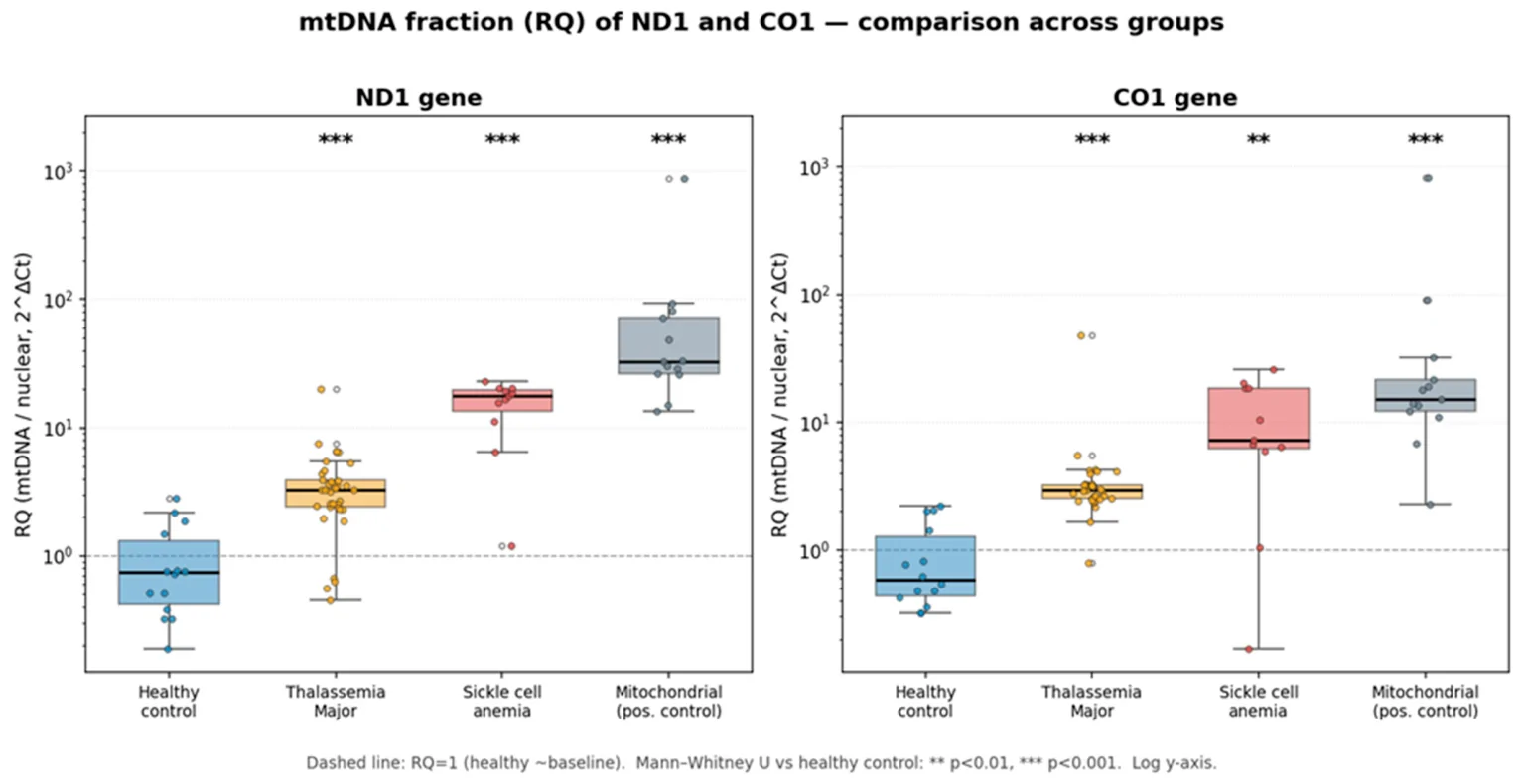
Box plot of ND1 and CO1 mtDNA fraction (RQ) by group. Dashed line marks RQ = 1 (healthy ~ baseline); the y-axis is logarithmic. Versus healthy control: ** *p* < 0.01, *** *p* < 0.001.

The quantity of total plasma cfDNA and its relative content of cf-mtDNA were measured in 36 β-thalassemia patients, in 11 SCD individuals, in 13 mitochondrial disease patients, and in 14 healthy individuals. cf-mtDNA content was also higher in all patient groups in relation to the healthy group (*p* = 0.008, Mann–Whitney U = 1591 and *p* < 0.001, Mann–Whitney U = 821, respectively). CO1 and ND1 relative abundance values were compared among the four study groups (Control, β-thalassemia major, sickle cell disease, and mitochondrial disease) (Figure 1). Since the data were not normally distributed, the Kruskal–Wallis test was used. Significant differences were observed for both CO1 and ND1 relative abundance values (both *p* = 0.0001) (Table 2). Dunn’s multiple-comparison post hoc test was subsequently performed to identify the group comparisons responsible for these differences, and the results are presented in Table 3.

A weak positive correlation was observed between the fraction of cf-mtDNA and total plasma cf-DNA in the β-thalassemia group (*p* = 0.04, r = 0.252).

### 3.3. Association of cf-mtDNA with Clinical Characteristics

Exploratory analyses were performed to assess potential clinical factors associated with cf-mtDNA levels. In the β-thalassemia group, neither ND1 nor CO1 levels were significantly correlated with serum ferritin, transfusion interval, liver iron concentration, or cardiac T2* values (all *p* > 0.05, Supplementary Table S1). ND1 levels were higher in splenectomized than in non-splenectomized patients (median 4.56 vs. 1.87, *p* = 0.042), whereas the corresponding difference in CO1 levels was not statistically significant (median 3.69 vs. 2.66, *p* = 0.130). In the SCD group, hydroxyurea use was not significantly associated with either ND1 or CO1 levels (*p* = 0.662 and *p* = 0.247, respectively). These findings should be considered exploratory given the small subgroup sizes.

In the β-thalassemia group, no significant correlations were observed between cf-mtDNA levels and hemoglobin, hematocrit, or venous blood gas parameters. For ND1, the correlation coefficients were ρ = 0.094 (*p* = 0.597) for hemoglobin, ρ = 0.072 (*p* = 0.684) for hematocrit, ρ = −0.032 (*p* = 0.856) for pO_2_, and ρ = −0.030 (*p* = 0.864) for sO_2_. Similarly, CO1 was not significantly correlated with hemoglobin (ρ = 0.068, *p* = 0.701), hematocrit (ρ = 0.025, *p* = 0.888), pO_2_ (ρ = 0.220, *p* = 0.212), or sO_2_ (ρ = 0.174, *p* = 0.326, Supplementary Table S1).

### 3.4. The Discriminatory Performance of CO1 and ND1 (ROC Curve Analysis)

To evaluate the diagnostic utility of CO1 and ND1, receiver operating characteristic (ROC) curve analysis was performed for each pairwise group comparison. The area under the curve (AUC), optimal cut-off value, sensitivity, specificity, positive predictive value (PPV), negative predictive value (NPV), and positive likelihood ratio (LR+) were calculated for each marker; an AUC greater than 0.70 was considered the threshold for acceptable discrimination. Because PPV and NPV are dependent on disease prevalence, and the group sizes in this case-control design were fixed by study recruitment rather than reflecting population prevalence, the PPV and NPV values reported below should be interpreted as descriptive of this study sample and are not directly generalizable to the broader clinical population.

**Figure 2:**
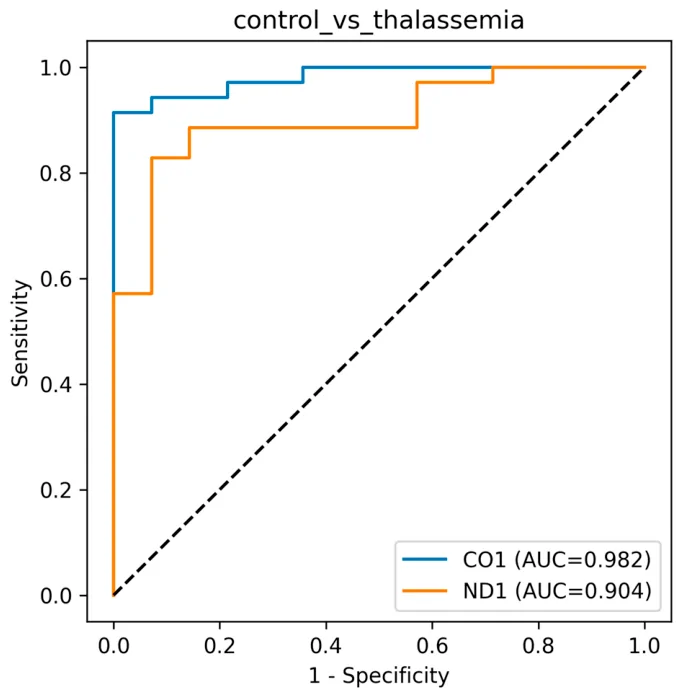
ROC curves of CO1 and ND1 for discrimination between healthy controls and patients with β-thalassemia major.

ROC analysis demonstrated excellent diagnostic performance for both biomarkers in distinguishing β-thalassemia major from healthy controls, with CO1 achieving an AUC of 0.982 (95% CI: 0.894–0.996) and ND1 an AUC of 0.904 (95% CI: 0.785–0.969) (Table 4, Figure 2 and Supplementary Table S2). At the optimal cut-off of >2.17, CO1 reached 91.43% sensitivity and 92.86% specificity (LR+ = 12.80).

**Figure 3:**
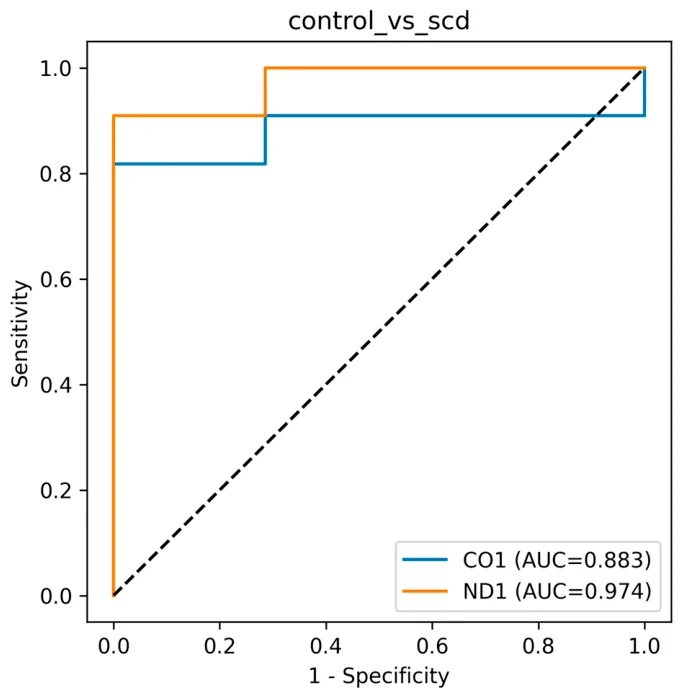
ROC curves of CO1 and ND1 for discrimination between healthy controls and patients with sickle cell disease.

For sickle cell disease versus healthy controls, ND1 showed excellent discriminatory ability (AUC = 0.974, 95% CI: 0.817–0.990), while CO1 showed good performance (AUC = 0.883, 95% CI: 0.791–0.974) (Table 5, Figure 3). At a cut-off of >2.19, ND1 achieved 90.91% sensitivity and 71.43% specificity (LR+ = 3.18).

## 4. Discussion

In hemoglobinopathies such as β-thalassemia and sickle cell disease (SCD), chronic hemolysis, oxidative stress, and inflammation are central to disease pathogenesis. Mitochondrial dysfunction has also been implicated in these disorders, and cf-mtDNA is increasingly recognized both as a marker of mitochondrial damage and a proinflammatory DAMP [5,6,7,8]. In our study, the relative abundance of plasma cf-mtDNA was significantly higher in patients with β-thalassemia and SCD than in healthy controls. A similarly increased relative cf-mtDNA abundance was observed in patients with primary mitochondrial disorders, supporting the interpretation that mitochondrial damage may contribute to the circulating cf-mtDNA signal observed in hemoglobinopathies.

Although cf-mtDNA dynamics have been extensively investigated in SCD, their role in β-thalassemia remains less well understood. A recently published study reported increased mitochondrial DNA damage in transfusion-dependent β-thalassemia patients—associated with systemic iron overload highlighting the biomarker’s potential prognostic relevance [13]. Additionally, the responsiveness of cf-mtDNA to diverse stressors, including physiological, psychological, and inflammatory stimuli, has been demonstrated in human studies, suggesting significant intra-individual variability and a complex regulatory profile [12].

In SCD, elevated cf-mtDNA/cf-nDNA ratios have been associated with NET formation and persistent inflammation [10,14]. This process may be related to mitochondrial retention in mature erythrocytes, an aberrant phenomenon that provides a direct source of cf-mtDNA. Retained mitochondria have also been shown to promote excessive ROS generation and mitochondrial stress [10,11].

In β-thalassemia, particularly in transfusion-dependent patients, iron overload further exacerbates mitochondrial injury by promoting ROS production and impairing mitophagy. Deletion mutations such as ΔmtDNA4977 have been reported at higher frequencies in these patients, indicating cumulative mitochondrial damage [13]. Oxidized hemoglobin derivatives (e.g., methemoglobin, ferry hemoglobin), along with free heme and iron, can disrupt mitochondrial membranes and facilitate cf-mtDNA release [3,9]. These oxidized components also act as DAMPs and contribute to endothelial dysfunction and systemic inflammation [5]. While standard therapies include regular transfusions, iron chelation, and hydroxyurea, recent evidence suggests that mitochondrial-targeted nutritional supplementation such as coenzyme Q10, riboflavin, and L-carnitine may mitigate oxidative injury and preserve mitochondrial function [9].

One potential mechanism that could contribute to the higher relative cf-mtDNA abundance observed in β-thalassemia involves altered mitochondrial clearance during erythroid maturation. Studies of erythroid precursors from patients with β^0^-thalassemia/HbE have demonstrated increased BNIP3L and PINK1 expression despite delayed maturation and persistent mitochondrial retention [15]. These findings suggest that activation of mitophagy-related pathways may be insufficient to fully compensate for mitochondrial stress in affected erythroid cells. Although mitophagy was not assessed in our cohort, impaired or incomplete mitochondrial clearance could hypothetically contribute to the increased circulating mitochondrial DNA signal observed in β-thalassemia.

In SCD, altered mitochondrial clearance may provide another potential mechanism contributing to circulating cf-mtDNA. Previous studies have reported mitochondrial retention in mature erythrocytes, suggesting incomplete mitophagy during erythroid maturation [16]. Retained mitochondria may contribute to increased ROS generation and could provide a source of mitochondrial material following hemolysis. Although these observations provide a biologically plausible explanation for increased circulating mitochondrial DNA in SCD, mitophagy and mitochondrial retention were not directly assessed in our cohort, and this mechanism therefore remains hypothetical in relation to our findings.

The potential inflammatory consequences of cf-mtDNA release may also be relevant to hemoglobinopathies. In the SCD cohort studied by Tumburu and colleagues, inhibition of TBK1, a signaling kinase downstream of the cGAS–STING pathway, reduced mtDNA-associated neutrophil extracellular trap formation [10]. These findings raise the possibility that circulating mtDNA may contribute to inflammatory signaling through cytosolic DNA-sensing pathways. However, neither cGAS–STING activation nor NETosis markers were measured in the present study, and therefore any relationship between the higher relative cf-mtDNA abundance observed in our patients and these pathways remains hypothetical. Whether cf-mtDNA specifically, rather than total cfDNA, changes in response to therapy remains an open question. In our exploratory analysis, cf-mtDNA measures were evaluated in relation to hydroxyurea use in the SCD group; however, the small sample size limits meaningful inference regarding treatment effects. Similarly, transfusion-related variables and iron overload markers may influence circulating cf-mtDNA and should be considered potential confounders. Therefore, any relationship between cf-mtDNA and treatment response remains hypothetical and cannot be established from the present cross-sectional data. Prospective longitudinal studies incorporating treatment status, transfusion timing, and disease activity at the time of sampling will be required to determine whether cf-mtDNA changes in response to therapy and whether serial measurement has potential value for monitoring treatment response.

The choice of quantification and normalization strategy is important when interpreting cf-mtDNA measurements. In the present study, mitochondrial targets were assessed by SYBR Green-based qPCR relative to nuclear reference genes. Accordingly, the resulting values represent relative mitochondrial DNA abundance rather than absolute circulating mtDNA copy number. Importantly, an mtDNA:nDNA-based measure can be influenced by variation in either component; therefore, a higher relative value does not necessarily indicate an equivalent absolute increase in circulating mtDNA. Absolute quantification approaches, such as droplet digital PCR, may help clarify this distinction in future studies and facilitate comparison across cohorts and laboratories [17].

From a translational perspective, a noninvasive, blood-based biomarker such as cf-mtDNA holds appeal in pediatric hemoglobinopathy care, where repeated invasive sampling is best minimized. In our study, exploratory analyses of potential clinical modifiers showed no significant associations between cf-mtDNA levels and transfusion interval or markers of iron overload. Interestingly, ND1 levels were higher in splenectomized patients, whereas no significant difference was observed for CO1. Although altered clearance of circulating cellular and mitochondrial material following splenectomy may potentially contribute to this observation, the small subgroup size and lack of a consistent association across both cf-mtDNA targets preclude definitive conclusions. This finding should therefore be regarded as hypothesis-generating and requires confirmation in larger cohorts. If future longitudinal studies confirm that cf-mtDNA tracks with treatment response, serial measurement could complement existing monitoring tools such as serum ferritin, liver iron concentration, and transfusion requirement by providing a more immediate readout of mitochondrial and oxidative injury, one that may respond to intervention (e.g., intensified iron chelation or initiation of mitochondrial-targeted antioxidants) more rapidly than conventional iron-burden indices. Such an approach would be consistent with the broader trend toward incorporating DAMP-based biomarkers into precision monitoring strategies for chronic inflammatory and hemolytic disorders.

Several limitations of the present study should be acknowledged. First, the cross-sectional design precludes conclusions about the temporal relationship between cf-mtDNA release and clinical events such as vaso-occlusive crises or transfusion cycles; longitudinal sampling would be required to establish whether cf-mtDNA levels rise before, during, or after acute episodes. Second, the relatively modest subgroup sizes, particularly in the SCD group, limit statistical power for subgroup comparisons and increase the risk of type II errors for weaker associations. Third, this study used plasma exclusively rather than serum; because the two matrices are not strictly interchangeable for cell-free DNA quantification, since the clotting process itself can release additional nuclear and mitochondrial material, our findings should be considered specific to the plasma matrix rather than directly generalizable to serum-based assays, and future studies should explicitly compare the two specimen types. Fourth, cf-mtDNA measurements are particularly sensitive to pre-analytical factors, including blood collection, processing delay, centrifugation conditions, and residual cellular or platelet contamination, which may influence the measured mitochondrial DNA signal. Although venous pO_2_ and sO_2_ were evaluated as supportive clinical parameters, they do not directly reflect tissue oxygenation, and no significant association with cf-mtDNA levels was observed in the present study. Finally, mitophagy markers (e.g., BNIP3L, PINK1, LC3-II) and NETosis markers were not directly measured in the same patients, so the mechanistic links proposed above remain inferential rather than directly demonstrated in our cohort.

Addressing these points in future prospective, multicenter studies would strengthen the case for cf-mtDNA as a clinically actionable biomarker. For cf-mtDNA to be adopted as a routine clinical biomarker rather than remain a research tool, standardization of the measurement methodology will be a prerequisite. Differences across studies in specimen matrix (plasma versus serum), extraction protocol, choice of mitochondrial target (e.g., ND1, CO1, or other regions), normalization strategy, and quantification platform (qPCR versus digital PCR, discussed above) all affect the absolute values obtained, which currently limits direct comparison of cut-off values across laboratories and cohorts, including our own. Establishing a consensus, harmonized protocol ideally validated across multiple centers would be a necessary next step before cf-mtDNA thresholds such as those reported in Table 4 and Table 5 could be applied clinically. The ROC analyses should be interpreted as exploratory assessments of discriminatory performance rather than as evidence of established diagnostic utility. The relatively small sample size, particularly in the SCD and mitochondrial disease groups, and the absence of an independent validation cohort preclude definition of clinically applicable cut-off values. Prospective validation in larger, independent and longitudinal cohorts will therefore be required before diagnostic or monitoring thresholds can be proposed.

Before cf-mtDNA measurement can be translated into routine clinical practice, several analytical and clinical requirements need to be addressed. These include standardized pre-analytical procedures, analytical validation of the assay, appropriate internal and external quality-control procedures, interlaboratory harmonization, and documented staff competency. Clinically validated reporting thresholds should be established in larger independent cohorts, together with clear guidance for interpretation of borderline, false-positive, and false-negative results. Cost-effectiveness and data-governance requirements should also be considered before routine implementation. Importantly, cf-mtDNA should not be used as a stand-alone diagnostic marker until its clinical performance and decision thresholds have been prospectively validated; interpretation should remain integrated with established clinical and laboratory findings, with responsibility for diagnostic decisions remaining within the appropriate clinical and laboratory governance framework [18].

In conclusion, cf-mtDNA may represent a potential biomarker of mitochondrial dysfunction in hemoglobinopathies such as β-thalassemia and SCD. The observed higher relative cf-mtDNA abundance is consistent with increased mitochondrial stress and damage in these disorders. Validation of these findings in larger, prospective and longitudinal studies is needed to better define the pathophysiological significance and potential clinical utility of cf-mtDNA, including its possible role in disease monitoring and assessment of therapeutic response.

## 5. Conclusions

Overall, the findings of this study indicated that circulating cf-mtDNA was significantly elevated in patients with β-thalassemia and sickle cell disease compared with healthy controls, consistent with increased mitochondrial stress or injury in hemoglobinopathies. In both β-thalassemia major and SCD, the relative abundance of the mitochondrial targets ND1 and COI was significantly increased compared with healthy controls. The mitochondrial disease group, included as a positive control, showed the highest values, supporting the expected direction of the findings. These results suggest that cf-mtDNA may have potential as a noninvasive, indirect biomarker associated with mitochondrial stress in inherited hemolytic disorders. However, its potential utility for disease monitoring or assessment of therapeutic response requires validation in larger, prospective longitudinal studies.

## Figures and Tables

**Table 1:** Demographic characteristics of the study population.

Characteristic	Control (*n* = 14) Mean ± SD	β-Thalassemia Major (*n* = 36) Mean ± SD,	Sickle Cell Disease (*n* = 11) Mean ± SD	Mitochondrial Disease (*n* = 13) Mean ± SD	*p* Value
Age (years), mean ± SD	23.79 ± 8.60	23.81 ± 10.04	17.73 ± 8.77	17.08 ± 9.24	0.076 *
Male, *n* (%)	6 (42.86)	21 (58.33)	7 (63.64)	7 (53.85)	0.722 †
Female, *n* (%)	8 (57.14)	15 (41.67)	4 (36.36)	6 (46.15)	
Transfusion therapy, *n* (%)		36 (100)	—	—	
Chelation therapy, *n* (%)		36 (100)			
Hydroxyurea therapy, *n* (%)		—	5/11 (45.5)		
Splenectomy, *n* (%)		12/32 (37.5)			
Iron burden					
Ferritin, ng/mL		1486 (852–3096), *n* = 35	2118.5 (896.5–3079.5)		
Cardiac T2 *, ms		35.0 (33.0–42.9), *n* = 27	39.0 (37.4–40.7), *n* = 2		
Hematologic parameters					
Hemoglobin, g/dL		8.8 ± 1.2, *n* = 35	8.7 ± 1.2, *n* = 11		
Hematocrit, %		26.4 ± 3.8, *n* = 35	25.9 ± 3.9, *n* = 11		
LDH, U/L		235 (186–318), *n* = 32	366 (291–436), *n* = 10		
Oxygenation parameters					
pO_2_, mmHg		44.4 (36.1–57.0), *n* = 36	44.0 (34.5–57.2), *n* = 9		
sO_2_, %		78.5 (62.1–87.6), *n* = 36	72.7 (55.9–89.2), *n* = 9		

* One-way ANOVA. † Chi-square test. No statistically significant differences were observed among the four study groups with respect to age (*p* = 0.076) or sex distribution (*p* = 0.722). Data are presented as mean ± SD, median (interquartile range), or *n* (%), as appropriate. Sample sizes may vary across individual parameters because of missing clinical data. Disease-specific variables are reported only for the relevant patient groups.

**Table 2:** Comparison of CO1 and ND1 relative abundance among study groups.

Group	CO1 Mean ± SD	CO1 Median (IQR)	ND1 Mean ± SD	ND1 Median (IQR)
Control	0.91 ± 0.70	0.58 (0.40–1.57)	0.97 ± 0.80	0.74 (0.37–1.57)
β-Thalassemia Major	4.27 ± 7.63	2.95 (2.49–3.26)	3.74 ± 3.29	3.26 (2.38–3.94)
Sickle Cell Disease	11.02 ± 8.44	7.22 (5.99–18.59)	15.47 ± 6.58	17.83 (11.28–20.16)
Mitochondrial Disease	82.48 ± 221.62	15.09 (11.64–26.73)	106.34 ± 234.45	32.67 (26.06–76.66)

Kruskal–Wallis overall comparison: CO1, *p* = 0.0001; ND1, *p* = 0.0001.

**Table 3:** Pairwise comparisons of CO1 and ND1 levels (Dunn’s multiple-comparison test).

Comparison	CO1 (*p*)	ND1 (*p*)
Control vs. β-Thalassemia Major	0.0001	0.0001
Control vs. Sickle Cell Disease	0.001	0.0001
Control vs. Mitochondrial Disease	0.0001	0.0001
β-Thalassemia Major vs. Sickle Cell Disease	0.003	0.0001
β-Thalassemia Major vs. Mitochondrial Disease	0.0001	0.0001
Sickle Cell Disease vs. Mitochondrial Disease	0.037	0.001

**Table 4:** The discriminatory performance of CO1 and ND1 for discriminating β-thalassemia major from healthy controls.

Marker	AUC	95% CI	Cut-Off	Sensitivity (%)	Specificity (%)	PPV (%)	NPV (%)	LR(+)
CO1	0.982	0.894–0.996	>2.17	91.43	92.86	97.00	81.27	12.80
ND1	0.904	0.785–0.969	>2.18	82.86	92.86	96.78	68.45	11.60

**Table 5:** The discriminatory performance of CO1 and ND1 for discriminating sickle cell disease from healthy controls.

Marker	AUC	95% CI	Cut-Off	Sensitivity (%)	Specificity (%)	PPV (%)	NPV (%)	LR(+)
CO1	0.883	0.791–0.974	>2.05	81.82	71.43	69.23	83.37	2.86
ND1	0.974	0.817–0.990	>2.19	90.91	71.43	71.48	90.96	3.18

## Data Availability

The data presented in this study are available on request from the corresponding author, due to patient privacy considerations.

## Supplementary Materials

The following supporting information can be downloaded at: https://www.mdpi.com/article/10.3390/biomedicines14092011/s1, Table S1: Correlations between cf-mtDNA levels and clinical and laboratory parameters; Table S2: ROC analyses of cf-mtDNA markers.
