# Supplementary material for: Mitochondrial Damage in Hemoglobinopathies: The Role of Cell-Free mtDNA as a Potential Biomarker
Source: Biomedicines. 2026 Sep 7;14(9):2011. doi: 10.3390/biomedicines14092011 (PMC13604636; doi:10.3390/biomedicines14092011)
Supplement: Supplementary file 1 [file biomedicines-14-02011-s001.zip › biomedicines-4506927-supplementary.pdf]

Supplementary Tables

Supplementary Table S1. Correlations between cf-mtDNA levels and clinical and laboratory parameters

Clinical parameter	n	ND1 Spearman's ρ	p-value	n	CO1 Spearman's ρ	p-value
**Hemoglobinopathy cohort (β-thalassemia + SCD)**						
Hemoglobin (HGB)	45	−0.018	0.907	45	−0.041	0.790
ctHb	43	−0.023	0.884	43	−0.112	0.474
Pre-transfusion Hb	30	−0.137	0.472	30	−0.073	0.703
pO_2	43	0.036	0.819	43	0.283	0.066
sO_2	43	0.024	0.881	43	0.250	0.106
FO_2Hb	43	−0.016	0.918	43	0.251	0.104
ctO_2	43	−0.042	0.790	43	0.215	0.166
**β-thalassemia subgroup**						
Ferritin	34	−0.168	0.342	34	−0.279	0.111
Transfusion interval	31	−0.096	0.608	31	0.214	0.248
Liver iron concentration (MRI)	19	0.293	0.223	19	−0.021	0.932
Cardiac T2* (MRI)	26	−0.258	0.203	26	−0.036	0.863

Note. Data are presented as Spearman's rank correlation coefficients (ρ). Correlations were calculated using available cases; therefore, sample sizes varied according to the availability of clinical data. No statistically significant correlations were observed (all $p > 0.05$). * One-way ANOVA.

Supplementary Table S2. ROC analyses of cf-mtDNA markers

Comparison	Marker	Cases / Controls	AUC (95% CI)	Cutoff	Sensitivity, % (95% CI)	Specificity, % (95% CI)
Control vs β-thalassemia	CO1	35 / 14	0.982 (0.894–0.996)	>2.17	91.43 (76.9–98.2)	92.86 (66.1–99.8)
	ND1	35 / 14	0.904 (0.785–0.969)	>2.18	82.86 (66.4–93.4)	92.86 (66.1–99.8)
Control vs SCD	CO1	11 / 14	0.883 (0.791–0.974)	>2.05	81.82 (48.2–97.7)	71.43 (41.9–91.6)
	ND1	11 / 14	0.974 (0.817–0.990)	>2.19	90.91 (58.7–99.8)	71.43 (41.9–91.6)
Control vs mitochondrial disease	CO1	13 / 14	1.000 (0.871–1.000)	>2.20	100.00 (75.3–100.0)	100.00 (76.8–100.0)
	ND1	13 / 14	1.000 (0.871–1.000)	>2.80	100.00 (75.3–100.0)	100.00 (76.8–100.0)
β-thalassemia vs SCD	CO1	11 / 35	0.797 (0.733–0.901)	>5.48	81.82 (48.2–97.7)	97.14 (85.1–99.9)
	ND1	11 / 35	0.896 (0.770–0.966)	>6.43	90.91 (58.7–99.8)	91.43 (76.9–98.2)
β-thalassemia vs mitochondrial disease	CO1	13 / 35	0.908 (0.788–0.972)	>5.48	92.31 (64.0–99.8)	97.14 (85.1–99.9)
	ND1	13 / 35	0.996 (0.917–1.000)	>7.54	100.00 (75.3–100.0)	97.14 (85.1–99.9)
SCD vs mitochondrial disease	CO1	13 / 11	0.786 (0.687–0.872)	>10.45	84.62 (54.6–98.1)	63.64 (30.8–89.1)
	ND1	13 / 11	0.888 (0.793–0.977)	>15.00	84.62 (54.6–98.1)	27.27 (6.0–61.0)

Notes. AUC, area under the receiver operating characteristic curve; CI, confidence interval; SCD, sickle cell disease. Sensitivity and specificity confidence intervals were calculated using the exact binomial (Clopper–Pearson) method.
